# Annual patterns of airborne bacteria governed by local generation and regional dispersal

**DOI:** 10.1128/aem.01345-25

**Published:** 2026-01-28

**Authors:** So-Yeon Jeong, Chi Won Lee, Tae Gwan Kim

**Affiliations:** 1 Department of Microbiology, Pusan National University 34996, Pusan, Republic of Korea; Washington University in St. Louis, St. Louis, Missouri, USA

**Keywords:** airborne bacteria, local generation, dispersal, seasonality

## Abstract

Airborne bacteria represent a critical yet highly variable component of atmospheric ecosystems, driven by a complex interplay of local generation (LG) and regional dispersal (RD). Through extensive monitoring over 3 years (315 aerosol samples), we quantified airborne bacterial populations using quantitative PCR combined with high-throughput sequencing, revealing substantial fluctuations (2.8–5.8 log_10_ copy·m^−3^) characterized by dramatic spring peaks and summer minima. Strikingly, episodic dust events and PM_10_ spikes from distant arid regions thousands of kilometers away increased local bacterial abundance by 5.5- and 6.2-fold (*P* < 0.05), respectively. Correlation network analysis identified strong positive associations among bacterial abundance, desert PM_10_, and local PM_10_, alongside negative relationships with temperature, humidity, and precipitation. Time-series analyses further revealed robust, synchronized annual cycles for bacterial abundance, desert PM_10_, and local PM_10_ (*P* < 0.05), with parametric modeling capturing a 4-week lag between desert dust emissions and subsequent local microbial peaks. Structural equation modeling provided quantitative confirmation that both LG and RD significantly influenced airborne bacterial dynamics (*P* < 0.05), with RD predominating during peak spring dust storm activity. Collectively, our findings highlight the substantial role of transcontinental dust transport as a primary source of airborne bacteria, complementing and sometimes overshadowing LGs in determining atmospheric bacterial communities.

**IMPORTANCE:**

This study enhances understanding of how airborne bacterial populations vary throughout the year in Busan, South Korea, by analyzing both local generation and long-distance transport. Over 3 years of continuous monitoring, we observed consistent spring peaks in bacterial abundance, closely linked to dust storms originating in the arid regions of China and Mongolia. These dust events transport large quantities of particles carrying bacteria over thousands of kilometers, temporarily raising local airborne bacterial levels above typical background levels. The findings show that while bacteria are continuously emitted from local sources, regional dust transport can be the dominant driver, particularly during the spring dust storm season. This combination of local and regional influences results in complex seasonal cycles of bacterial abundance. Understanding how these processes interact is critical for predicting changes in air quality, evaluating potential health risks, and recognizing the ecological connections that link distant desert environments with downwind areas.

## INTRODUCTION

Airborne microorganisms originate from diverse environments, disperse through the atmosphere, and ultimately deposit into new habitats (1, 2), rendering them transient inhabitants of the air (3, 4). Among various microbial groups, including bacteria, archaea, fungi, protozoa, and algae, bacteria have received particular attention due to their abundance, long-distance transport capability, critical roles in biogeochemical cycles, and potential health impacts as pathogens or allergens (1, 2, 5–7).

The abundance of airborne microorganisms results from two primary processes: local generation (LG; microbial emission from and deposition to nearby surfaces such as soils, waters, vegetation, and anthropogenic structures) and regional dispersal (RD; transport of microbes from surrounding regions) (3, 8–10). Local emissions occur when microbes from surfaces become aerosolized by air currents (5, 11), whereas removal occurs through processes like precipitation and gravitational settling (12). RD involves the transport of microbes by air masses over long distances, affecting microbial abundance in downwind regions (8, 13). These two processes are strongly influenced by meteorological conditions (e.g., temperature, humidity, precipitation), air pollutants (e.g., metals, SO_2_, O_3_, NO_x_), and major environmental events (e.g., dust storms, wildfires, volcanic eruptions) (2, 5, 6, 14).

Asian dust is a prominent example of RD, carrying substantial microbial loads across vast distances. Dust particles originating from the arid regions of China and Mongolia can travel thousands of kilometers to downwind areas such as Korea and Japan (13, 15–17). Bacteria transported during these dust events can be up to an order of magnitude more abundant than those emitted locally, with particulate matter (PM) acting both as a transport vehicle and as a protective refuge for small microorganisms, including bacteria and archaea (13, 15, 18).

The atmosphere is arguably the most dynamic habitat for microorganisms, in contrast to more stable ecosystems such as soils and aquatic environments (5, 12, 19). Airborne bacterial abundance fluctuates substantially across time and space due to the complex and ever-changing environmental conditions (19, 20). Despite this variability, recurring seasonal patterns in airborne bacterial abundance have been documented globally—for example, in the United States (Cleveland, Detroit, and rural Oregon) and Northern Italy (9, 21, 22). These patterns suggest that environmental factors, particularly temperature and moisture, play a key role in driving the seasonal cycles of airborne bacteria from local sources.

Previous studies on airborne microbial dynamics have typically examined LG and RD as separate processes, often focusing on only one of these factors (9, 23). Moreover, much of the research has relied on short-term data sets (1 year or less) with irregular sampling intervals, primarily capturing seasonal or monthly variations (10, 15, 24, 25). These limitations hinder our understanding of longer-term microbial fluctuations, including both intra- and inter-annual variability. To address these gaps, we conducted a 3-year observational study to investigate the temporal patterns of airborne bacterial abundance and to disentangle the relative contributions of LG and RD. By applying advanced analytical approaches, including correlation network analysis, spectral analysis, time-series decomposition, and structural equation modeling (SEM), we aimed to provide a comprehensive, long-term perspective on how these two processes jointly shape airborne microbial populations.

## RESULTS

### Long-term monitoring of bacterial abundance

**Fig 1:**
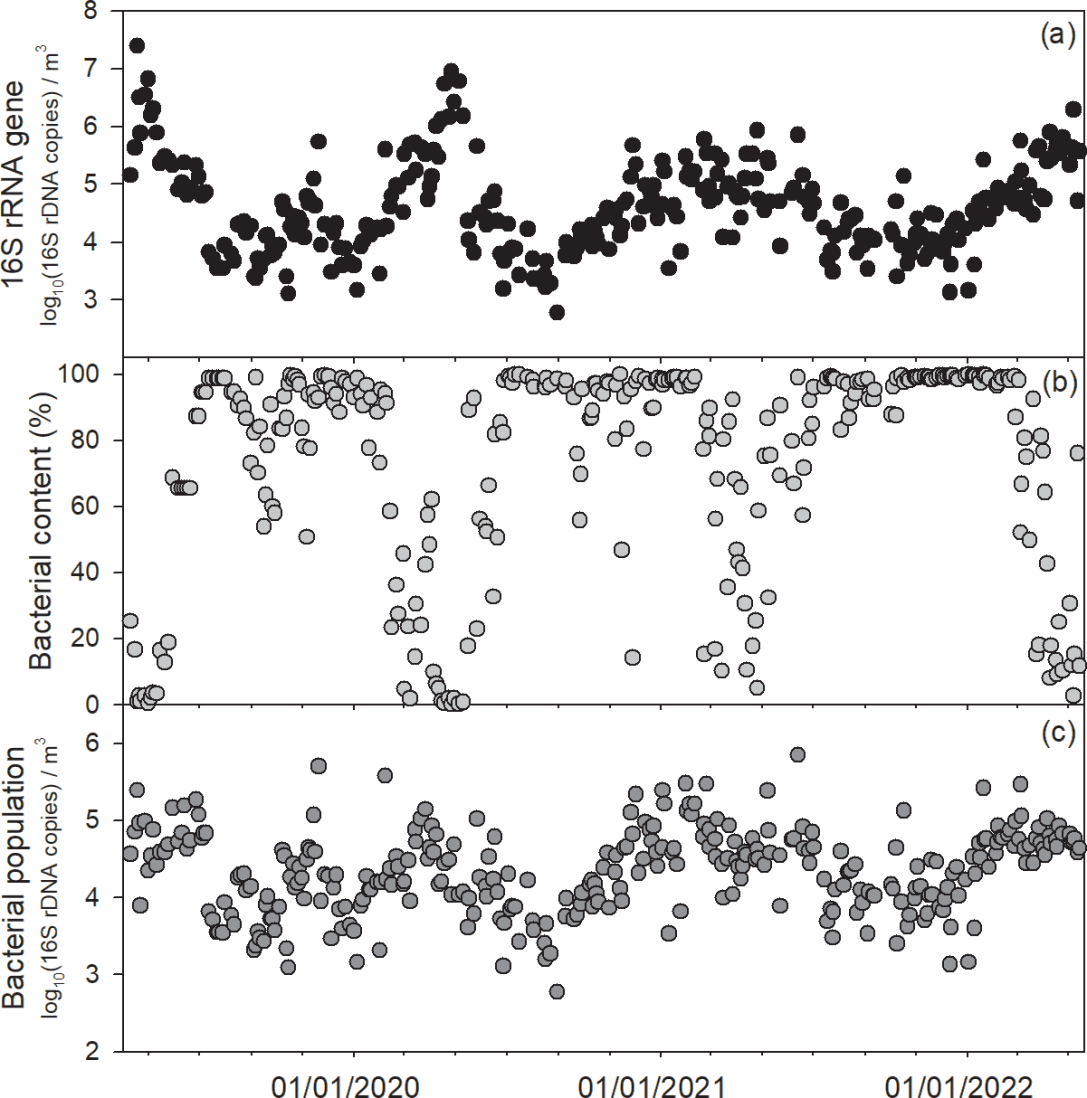
Temporal variation of airborne bacterial population over more than 3 years. (**a**) Absolute quantity of 16S rRNA gene; (**b**) bacterial content counted by parallel sequencing; and (**c**) bacterial population density adjusted by the 16S rRNA gene copy number and bacterial content.

Over the 3-year sampling period in Busan, South Korea, the 16S rRNA gene copy number ranged from 2.8 to 7.4 log_10_ copy·m^−3^, averaging 4.6 ± 0.8 log_10_ copy·m^−3^ (Fig. 1a). The rRNA population peaked consistently in spring (April–May) each year. Of the total 16S rRNA gene sequences, bacterial reads constituted 0.1%–100%, with an overall mean of 73.3% ± 33.0% (Fig. 1b). Non-bacterial sequences were predominantly chloroplast-derived (97.3% ± 7.2%). Notably, bacterial relative abundance dropped sharply in April (20.6% ± 13.5%) and May (38.0% ± 24.5%), compared to the rest of the year (85.1% ± 15.1%), when the data were aggregated monthly over the 3-year period.

When adjusted to exclude chloroplast and other non-bacterial sequences, the bacterial population (i.e., bacterial 16S rRNA gene copy numbers) ranged from 2.8 to 5.8 log_10_ copy·m^−3^, with a mean of 4.3 ± 0.5 log_10_ copy·m^−3^ (Fig. 1c). In line with the total 16S rRNA gene observations, these bacterial counts also peaked annually during spring, indicating the strong seasonality in airborne bacterial abundance.

### Correlation network

**Fig 2:**
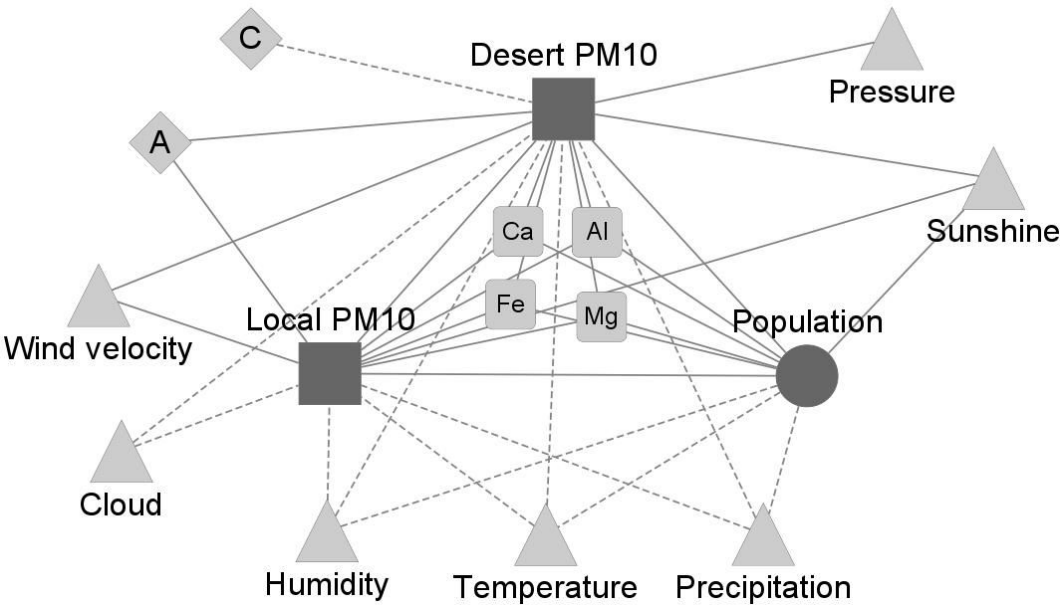
Correlation network of airborne bacterial population and environmental factors (e.g*.*, aerosol, air mass pathway, and meteorological factors). To emphasize the association of bacterial population with local PM_10_ and desert PM_10_, correlations between other environmental attributes were not included. Circles, squares, triangles, diamonds, and round squares represent bacterial population, PM, local meteorological factors, air mass pathways, and heavy metals, respectively. Letters A and C indicate the frequencies of air masses transported from the northwest and the southeast, respectively. Solid and dashed edges represent positive and negative correlations, respectively (*P* < 0.05).

A correlation-based network was constructed to examine the relationships among bacterial abundance, meteorological factors, and pollutants. Network module analysis revealed that bacterial abundance, local PM_10_ (Busan), and desert PM_10_ (e.g., Inner Mongolia and Gobi Desert) formed a cohesive module, exhibiting strong positive correlations. To further explore these associations, a subnetwork highlighting these three variables and their directly connected nodes was extracted (Fig. 2). In this subnetwork, population, local PM_10_, and desert PM_10_ all showed positive correlations with metals such as Ca, Mg, Al, and Fe, as well as with sunshine. By contrast, they were negatively associated with temperature, humidity, and precipitation. To determine air-mass origins, back-trajectory analyses were performed and grouped into five pathways (A–E) based on geographic source and transport direction (Fig. S1; e.g., pathway A: northern and mid-western China; pathway C: the North Pacific and southern Japan). Both local and desert PM_10_ were positively associated with pathway A, whereas desert PM_10_ showed a negative association with pathway C. These findings underscore the intertwined roles of local conditions and long-range dust transport in shaping airborne bacterial abundance.

### Annual variations in aerosols and environmental factors

**Fig 3:**
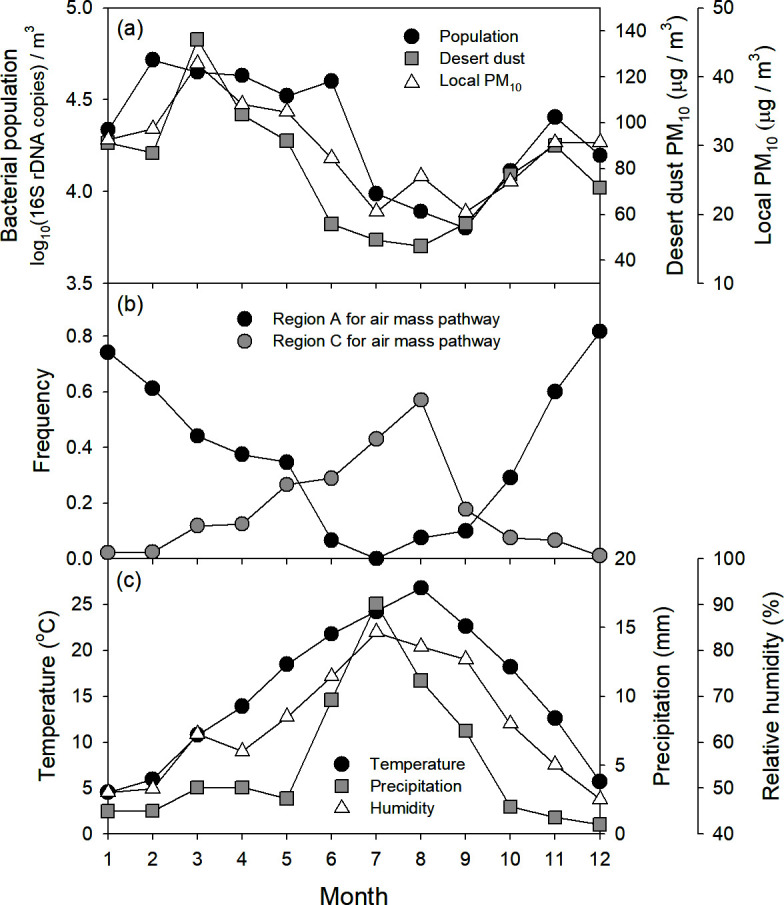
Seasonal variations of airborne bacterial population and environmental factors. (**a**) Airborne bacterial population and aerosol; (**b**) air mass pathway frequency; and (**c**) local meteorological factor.

To examine the annual patterns of aerosols and environmental factors, the data were aggregated on a monthly basis (Fig. 3). Bacterial population, desert PM_10_, and local PM_10_ followed broadly similar temporal patterns (Fig. 3a). Population was highest from February to June (4.6 ± 0.1 log_10_ copy‧m^−3^), reached its lowest level in September, and then rose again. Both desert PM_10_ and local PM_10_ peaked in March, declined to their minima in August–September, and subsequently increased. Air mass pathways A and C displayed differing annual trends (Fig. 3b). Pathway A formed a U-shaped curve, with its lowest frequency in July, whereas pathway C showed a hump-shaped pattern, peaking in August. Temperature, precipitation, and relative humidity each exhibited a hump-shaped trajectory (Fig. 3c), reaching their maxima in August, July, and July, respectively.

### Effects of long-range dust immigration on bacterial population

**Fig 4:**
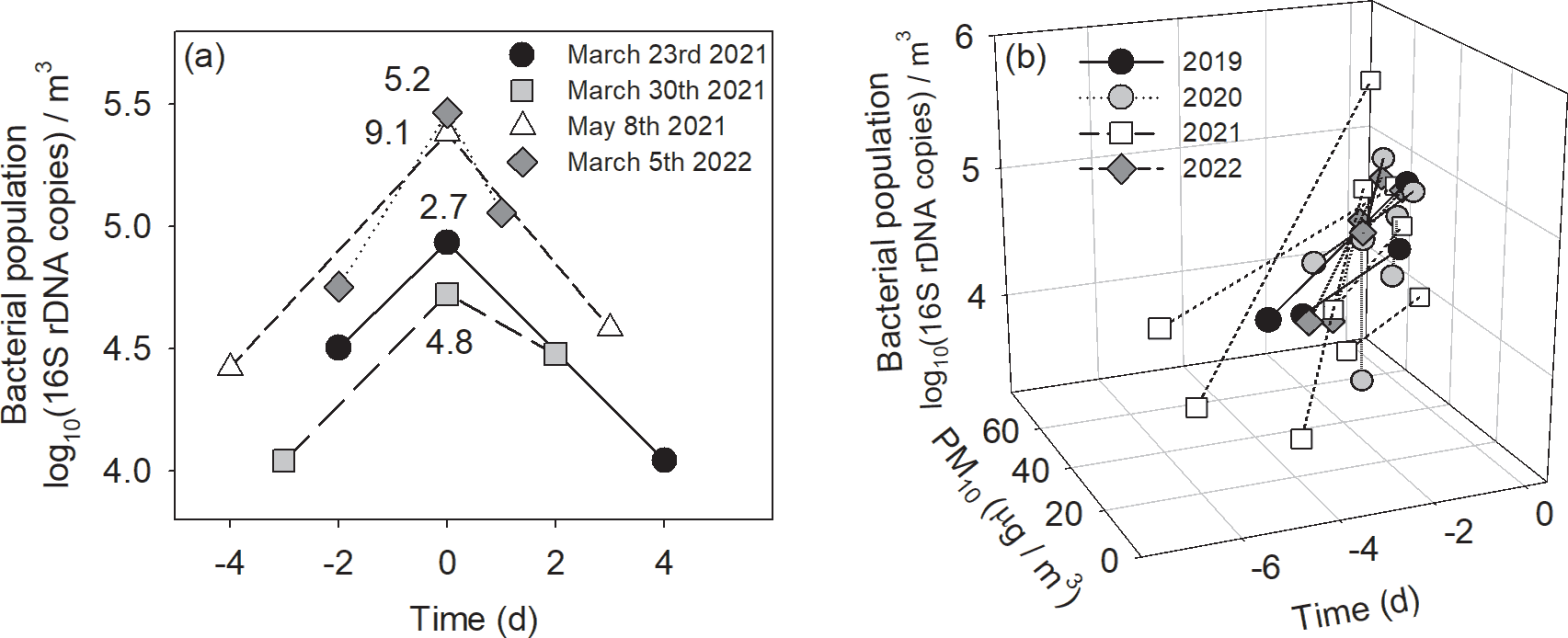
Effects of Asian dust event (**a**) and PM_10_ spiking (**b**) on airborne bacterial population. The numbers (5.2, 9.1, 2.7, and 4.8) indicate the ratio of the bacterial population during the Asian dust event to that before the event (**a**). Airborne bacterial population during a PM_10_ spike of more than 45.0 µg per m^3^ was compared to that before the spiking (the Asian dust events were excluded) (**b**).

To determine how Asian dust events influence local bacterial abundance, we compared airborne bacterial levels before, during, and after four distinct dust events recorded over the 3-year study (Fig. 4a). During these official dust events (declared by the KMA), PM_10_ concentrations rose from 33.8 ± 9.0 µg·m^−3^ to 121.0 ± 47.1 µg·m^−3^, then decreased to 39.5 ± 16.9 µg·m^−3^ afterward. Correspondingly, the bacterial population increased by 545% ± 268% (range: 271%–913%) compared with the pre-event levels (*P* < 0.05). After the events, it dropped by 69% ± 21% (range: 43–87%) relative to the event peaks (*P* < 0.05).

A similar trend emerged when PM_10_ concentrations spiked (i.e., > 45.0 µg·m^−3^) (Fig. 4b). Across 14 such instances, PM_10_ rose from 32.6 ± 15.8 µg·m^−3^ to 60.1 ± 9.5 µg·m^−3^, and the bacterial population surged by 622% ± 1,165% (range: 98%–4,500%) (*P* < 0.05). Collectively, these findings confirmed the substantial, though transient, increases in airborne bacterial abundance triggered by long-range dust transport from arid desert regions.

### Annual patterns of bacterial population, local PM_10_, and desert PM_10_

**Fig 5:**
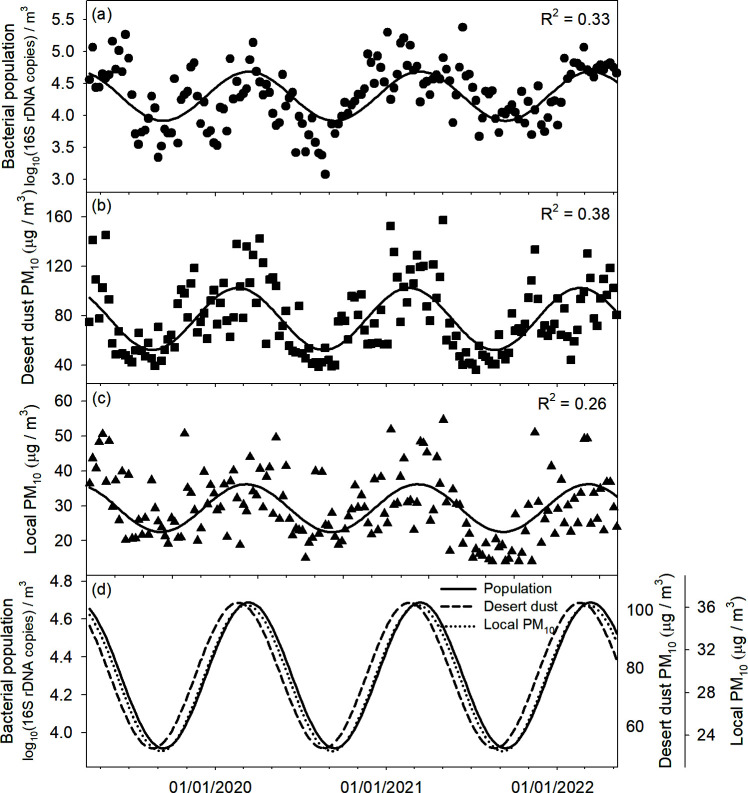
Comparison of seasonal patterns of airborne bacterial population (**a**), desert dust PM_10_ (**b**), and local PM_10_ (**c**). Symbols and lines represent observation (weekly data) and parametric simulation (*Y* = *a‧sin*(2‧π‧*t*) + *b‧cos*(2‧π‧*t*)), respectively. The seasonal simulations of airborne bacterial population, desert dust PM_10_, and local PM_10_ were compared (**d**).

Spectral analysis and time-series decomposition revealed that bacterial population, desert PM_10_, and local PM_10_ exhibited similar periodic trends, each peaking with an annual frequency (Fig. S3 to S6). To further characterize these annual cycles, the data were fitted to a sine/cosine model (*Y* = *a‧sin*(2‧π‧*t*) + *b‧cos*(2‧π‧*t*) (Fig. 5). The model accounted for 33%, 38%, and 26% of the variability observed in bacterial abundance, desert PM_10_, and local PM_10_, respectively (*P* < 0.05). Importantly, bacterial population and local PM_10_ showed nearly identical seasonal cycles, both consistently lagging approximately 4 weeks behind the desert PM_10_ pattern (Fig. 5d). This delay likely reflects the transit time required for dust and its associated microbial load to influence downwind environments.

### Structural equation model

**Fig 6:**
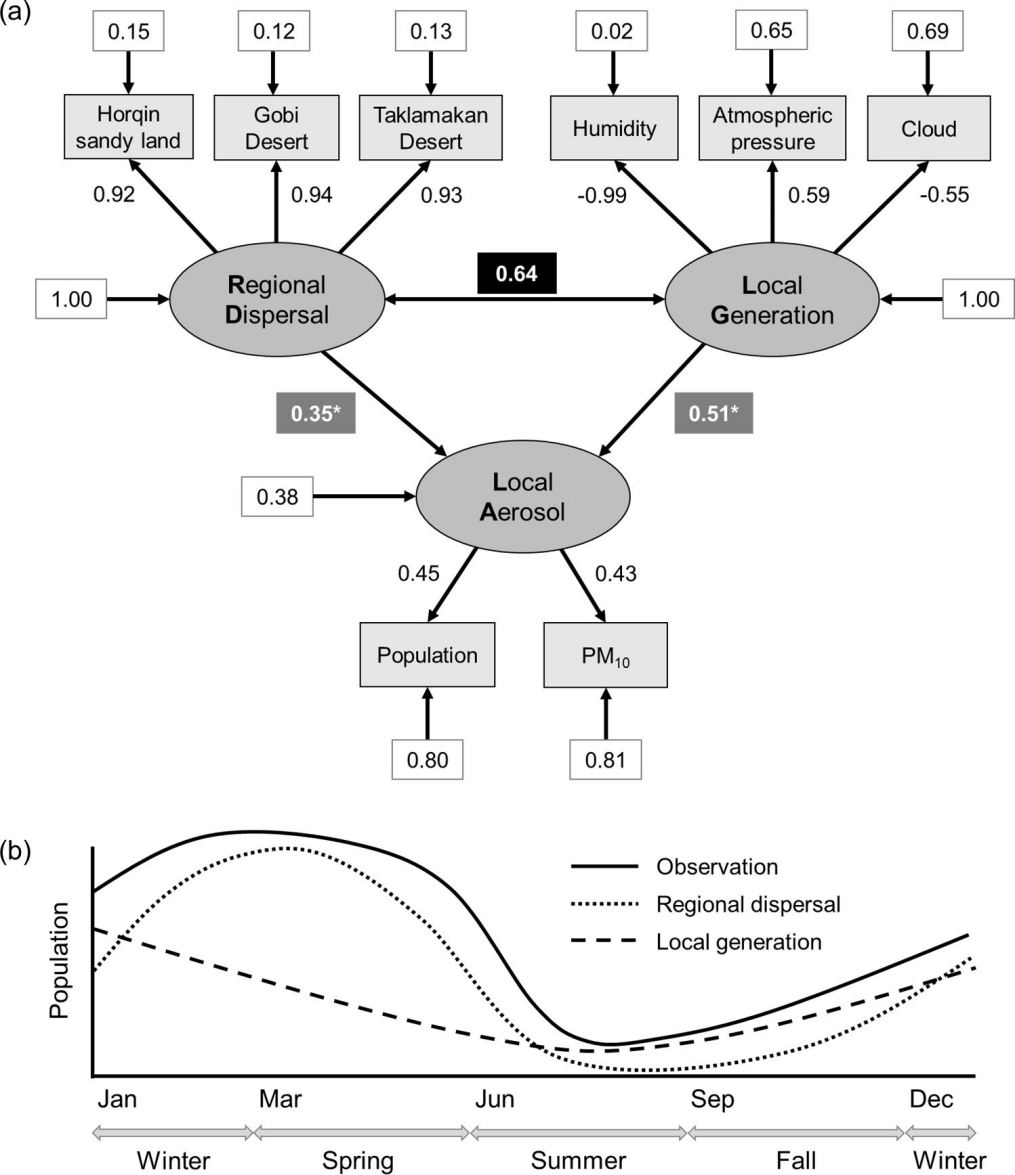
The conceptual and structural equation models of bacterial population dynamics in the atmosphere of southeastern Korea. The structural equation model depicts the assembly of LA through RD and LG (**a**). Shaded rectangles and ovals represent observed and latent variables, respectively. The number in the black rectangle represents the covariance between the latent variables. The number in the gray-shaded rectangles is the coefficient of the structural regression (* indicates statistical significance, *P* < 0.05). All loadings, variances, and paths were standardized. Based on the structural equation model, the conceptual framework illustrates the temporal dynamics of airborne bacterial populations, emphasizing their emergence through the combined influence of RD and LG (**b**).

To clarify how LG and RD together shape airborne bacterial population dynamics, we performed an SEM analysis using three latent variables: LG, RD, and local aerosol (LA) (Fig. 6a). The primary equation of the SEM model, LA = LG + RD, indicates that both LG and RD exert additive effects on LA. Overall, the model showed excellent fit with the data (Tucker-Lewis Index [TLI] = 0.963; root mean square error of approximation [RMSEA] = 0.083; standardized root mean square residual [SRMR] = 0.050). The latent variable LG was represented by relative humidity (path coefficient = –0.99; explained variance = 0.98), atmospheric pressure (0.59; 0.35), and cloud cover (–0.55; 0.31). RD was defined by three variables indicative of dust influx from desert regions in China and Mongolia, each showing high path coefficients (≥0.92) and substantial explained variance (≥0.85). LA was characterized by bacterial abundance (0.45; 0.20) and local PM_10_ concentration (0.43; 0.19). Both LG and RD significantly influenced LA, with standardized regression coefficients of 0.51 and 0.35, respectively (*P* < 0.05). Additionally, a positive covariance (0.64) between LG and RD implies that these two processes can co-occur and may interact synergistically, underscoring the complexity of airborne bacterial dynamics.

Building on these findings, we propose a conceptual model to illustrate how local airborne bacterial populations emerge from the interplay between LG and RD (Fig. 6b). In this framework, LG follows a U-shaped seasonal pattern (i.e., peaking in winter and reaching its minimum in summer) while RD through desert dust predominates between November and May, then declines sharply in summer. This dual influence underscores the complexity of airborne microbial dynamics, where both local conditions and distant dust transport collectively shape annual bacterial abundance.

## DISCUSSION

Over 3 years of observations in southeastern Korea, we documented a pronounced annual cycle in airborne bacterial abundance, strongly tied to Asian dust storms originating thousands of kilometers away. Our findings show that both LG and RD drive these bacterial fluctuations, with long-range dust transport often outweighing local sources, offering deeper insight into the temporal dynamics and source contributions of airborne microbes.

A total of 315 aerosol samples were collected during the 3-year sampling period in Busan, South Korea (Fig. 1). Quantitative PCR (qPCR) analysis revealed that 16S rRNA gene abundance varied markedly, from 2.8 to 7.4 log_10_ copy·m^−3^, highlighting substantial temporal fluctuations. The sequencing results further indicated that bacterial reads dominated the overall 16S rRNA gene libraries (73.3% on average), while most non-bacterial sequences consisted of chloroplast DNA (97.3% on average), indicating a considerable presence of phototrophic organisms in the atmosphere. This high proportion of chloroplast sequences aligns with reports from Colorado (USA) and Beijing (China), where chloroplast DNA was also detected in atmospheric samples (24, 26). Notably, our data showed that chloroplast sequences were especially prevalent in April (78.4%) and May (61.4%), compared with the other months (14.6%), likely due to heightened plant pollination during spring (27, 28). These findings underscore the need to account for chloroplast and mitochondrial DNA when using universal 16S rRNA primers to quantify airborne bacteria. This seasonal chloroplast influx also suggests a possible influence of pollen-associated microbes on airborne bacterial estimates. However, regression analysis between chloroplast DNA and bacterial 16S rRNA gene abundance yielded a very low R² value (0.01) (data not shown), indicating a minimal effect on our abundance estimates. After adjusting for non-bacterial sequences, bacterial abundance ranged from 2.8 to 5.8 log_10_ copies·m^−3^, with an average of 4.3 ± 0.5 log_10_ copies·m^−3^ (Fig. 1c). The clear annual peaks in spring highlight the strong seasonality of airborne bacterial abundance.

A correlation-based network analysis was conducted to examine the interrelationships among bacterial abundance, atmospheric contaminants, and meteorological variables. The resulting network revealed that bacterial abundance, local PM_10_, and desert PM_10_ were closely associated within the same module, indicating strong interconnections among them (Fig. 2). Given that airborne bacterial cells (< 3 µm) often occur as single cells, small aggregates, or dust-attached particles, their effective aerodynamic size falls largely within the coarse mode (2.5–10 µm) (19). Consequently, PM_10_ more accurately represents bacteria-laden particles, whereas PM_2_._5_ captures only a small fraction of intact or dust-associated cells. These three variables also showed positive correlations with soil-derived metals such as Ca, Mg, Al, and Fe (29, 30), suggesting that long-distance dust transport may carry both metals and bacterial cells from arid Asian dust sources to the study area. Notably, both local PM_10_ and desert PM_10_ were linked to air mass pathway A, the route of dust-laden air traveling southeastward. These findings are consistent with previous studies documenting the transboundary dispersal of Asian dust and its microbial content across East Asia, including China, Korea, and Japan (13, 17, 31). Local meteorological conditions also appeared to influence airborne bacterial abundance and PM_10_ levels, as all three variables were negatively correlated with temperature, humidity, and precipitation. Together, these findings suggest that the temporal dynamics of airborne bacteria in southeastern Korea are shaped by the combined effects of long-range dust transport and local environmental factors.

Monthly data aggregation revealed that bacterial abundance, desert PM_10_, and local PM_10_ followed parallel annual cycles, peaking in spring and declining in summer (Fig. 3a). This shared pattern suggests that long-range transport of particulates (including bacteria) from desert regions strongly influences local bacterial levels. Indeed, Asian dust production typically peaks in spring, driven by favorable wind patterns and dry soil conditions (32, 33). Reflecting this, air mass pathway A showed a U-shaped frequency curve, with its lowest occurrence in summer. Consequently, the influx of PM_10_ and bacteria also reached its highest level in spring and dropped to a minimum in summer, rebounding after summer as atmospheric conditions again favored dust production and transportation. Meanwhile, key meteorological factors, including temperature, precipitation, and humidity, which influence local bacterial generation (2, 7) exhibited hump-shaped patterns, peaking in summer (Fig. 3). Although elevated moisture and heat can enhance bacterial growth in soils and water (7, 34), they also promote wet deposition, reducing aerosol levels. In contrast, the drier winter conditions may favor microbial aerosolization from plants, soils, and waters, as frozen or snow-covered habitats are relatively rare in the study region. Thus, local bacterial generation appears to follow its own U-shaped pattern, further modulating atmospheric bacterial levels. Overall, these findings indicate that both regional dust transport and LG drive airborne bacterial abundance, with RD often exerting the stronger influence.

Asian dust originating from deserts in China and Mongolia can travel thousands of kilometers to downwind regions such as Korea and Japan (13, 17). These dust storms can substantially increase airborne microbial abundance in affected regions (13, 15). In this study, we evaluated the influence of long-range dust transport by tracking bacterial population shifts during both Asian dust events and PM_10_ spikes (Fig. 4). During official Asian dust events (https://www.kma.go.kr), local PM_10_ concentrations climbed from 33.8 to 121.0 µg·m^−3^, accompanied by a 545% surge in bacterial abundance. Once these events ended, both PM_10_ and bacterial numbers declined substantially, with abundance dropping by 69% relative to peak levels. A comparable pattern was observed during PM_10_ spikes linked to air mass pathway A, where PM_10_ rose from 32.6 to 60.1 µg·m^−3^ and bacterial counts increased by 622%. Together, these results confirm that Asian dust events deliver a temporary but substantial influx of particulates and microbes to distant regions. Interestingly, PM_10_ and PM_2_._5_ concentrations did not consistently correlate with bacterial abundance throughout the entire observation period (R^2^ ≤ 0.054, data not shown), suggesting that dust from arid areas can episodically, rather than uniformly, boost microbial loads. During 2019–2022, annual PM_10_ concentrations in Busan declined from 43.9 to 25.8 μg·m^–3^, consistent with reduced anthropogenic emissions during the COVID-19 period. In contrast, the airborne bacterial population showed a slight year-to-year increase despite the marked reduction in local PM_10_ (Fig. S4 and S5). Throughout the study period, long-range transport of Asian dust persisted and intermittently delivered substantial external particulate loads; these intrusions corresponded with seasonal increases in bacterial abundance. Collectively, these trends indicate that airborne bacterial populations were driven primarily by dust production in Asian desert source regions rather than by local anthropogenic activity. Moreover, to evaluate the contribution of long-range transport, the dominant taxa detected in Busan were directly compared with abundant taxa previously reported from Asian dust source regions, particularly the Gobi and Taklimakan Deserts. Table S1 summarizes the abundant bacterial taxa (16 families and 30 genera) detected in the atmosphere during Asian dust events and PM_10_ spikes. At the family level, 9 of 16 families overlapped with a source-region data set, including Acetobacteraceae, Bacillaceae, Chitinophagaceae, Geodermatophilaceae, Micrococcaceae, Moraxellaceae, Planococcaceae, Rhodobacteraceae, and Sphingomonadaceae (Fig. S2) (35). Genus-level comparisons likewise showed substantial correspondence, with 9 of 24, 12 of 28, and 4 of 7 genera shared across the three reference data sets (36–38) (Fig. S2). Among the shared genera, *Bacillus* and *Blastococcus* were detected in all three reference data sets, while *Rubellimicrobium*, *Nocardioides*, and *Kocuria* appeared in two of the three, all of which were included among the top 30 genera in our samples. These overlapping taxa are well documented as common inhabitants of arid soils and desert air masses, consistent with their frequent detection in dust-source environments (39, 40). These recurring desert-associated lineages strengthen the inference that long-range atmospheric transport is a major driver shaping the airborne bacterial community during dust-influenced periods. Together, these findings demonstrate that both acute dust events and persistent background inputs from desert source regions are key drivers shaping microbial abundance in downwind atmospheric environments.

RD associated with Asian dust significantly influenced airborne bacterial abundance in the study area. Spectral analysis of bacterial population, desert PM_10_, and local PM_10_ revealed similar peaks at a 1-year frequency (Fig. S3), indicating a strong annual cycle likely governed by regional climate patterns such as wind shifts, precipitation, and temperature (2). When modeled with sine/cosine functions, these cyclical behaviors accounted for 26%–38% of the total variance (*P* < 0.05; Fig. 5). While bacterial population and local PM_10_ traced nearly identical cycles, both lagged by about 4 weeks behind desert PM_10_ (Fig. 5d). This temporal lag likely corresponds to the time required for dust particles and associated microorganisms to travel and disperse from desert source regions to the study site (23). Such timing supports a cause-and-effect scenario, wherein desert PM_10_ influx spurs subsequent rises in local PM_10_ and bacterial counts. These findings highlight the ecological connectivity between distant desert ecosystems and downwind microbial communities, reinforcing previous observations that dust-associated microorganisms can significantly influence regional microbial dynamics (41). Ultimately, the observed temporal alignment and lag provide valuable insights into how RD shapes atmospheric bacterial population dynamics.

SEM has become a powerful tool for evaluating complex, multivariate causal relationships in ecology and environmental research (42, 43). In the context of airborne microbial communities, SEM is particularly valuable because bacterial abundance can be influenced by both LG (e.g., aerosolization from soil or water surfaces) and RD (e.g., long-range dust transport from arid regions) processes that are challenging to measure independently. In this study, we employed SEM to identify how LG and RD jointly shape LA levels (i.e., airborne bacterial populations and local PM_10_). We generated three latent variables—LG, RD, and LA—based on observed indicators that reflect meteorological conditions, dust immigration, and bacterial/PM_10_ measurements, respectively (Fig. 6a). The model exhibited robust fit indices (TLI = 0.963, RMSEA = 0.083, SRMR = 0.050), confirming its suitability for capturing the impacts of local and regional factors on atmospheric bacterial dynamics. Both LG and RD significantly contributed to LA, with regression coefficients of 0.51 and 0.35, respectively (*P* < 0.05). Their positive covariance (0.64) implies that LA production can coincide or even synergize with dust-driven dispersal events, reflecting the inherently multifaceted nature of atmospheric microbial dynamics. Building on these findings, we propose a conceptual framework that illustrates how LG follows a U-shaped seasonal pattern, peaking in winter and hitting its lowest levels in summer (Fig. 6b). During winter, low humidity but non-freezing conditions are conducive to aerosolization, allowing microbes from soil, water, and plant surfaces to become airborne (21, 44). By contrast, higher humidity and rainfall in summer promote wet deposition, reducing aerosol loads despite potentially higher microbial growth rates in the environment. In parallel, RD of dust-borne bacteria is most pronounced between November and May, likely tied to prevailing wind patterns and drier conditions in arid-source regions. During peak dust seasons, RD dominates local influences, temporarily masking the effects of LG. Overall, the SEM highlights the dynamic interplay of local and regional processes in shaping airborne bacterial communities. As climate change and land-use alterations continue to affect dust emissions and meteorological patterns, understanding this interplay will be critical for forecasting future trends in air quality, microbial exposure, and broader ecological impacts.

This 3-year observational study employed qPCR and high-throughput sequencing to investigate the seasonality of airborne bacterial abundance and to distinguish the relative contributions of LG and RD. The results revealed pronounced seasonal variation in bacterial abundance, fluctuating by over three orders of magnitude and strongly associated with Asian dust transported from desert regions in China and Mongolia, thousands of kilometers away. Both bacterial abundance and local PM_10_ exhibited synchronized annual cycles, consistently lagging approximately 4 weeks behind dust generation in the source deserts. SEM showed that both LG and RD significantly influenced airborne bacterial abundance, with regional dust-driven dispersal exerting a stronger effect, particularly during peak dust periods. These findings highlight the ecological connectivity between remote desert environments and urban atmospheres, demonstrating the profound impact of long-range aerosol and microbial transport on regional air quality. To our knowledge, this is the first comprehensive long-term study to characterize annual airborne bacterial dynamics and to elucidate the combined effects of local and regional processes using extensive atmospheric monitoring and quantitative modeling.

## MATERIALS AND METHODS

### Study area and sampling site

The study was conducted in Busan, South Korea (35°10′N 129°04′E), a metropolitan city with a temperate climate and four distinct seasons, characterized by hot, humid summers and cold, dry winters. The sampling site was situated at an altitude of 79 m on the campus of Pusan National University, located in a densely populated urban area with approximately 3.3 million residents and a total area of about 770 km^2^. According to the Korea Meteorological Administration (KMA, https://www.kma.go.kr), the average annual temperature and precipitation from 1991 to 2020 were 15°C and 1,577 mm, respectively.

### Aerosol collection

Two identical air sampling devices, as previously described by Jeong and Kim (45), were employed. Each device comprised a sterilized filter cup containing a 47 mm polyethersulfone membrane filter (0.2 µm pore size; SciLab Korea Co., Ltd., Seoul, Korea) connected to a vacuum rotary vane pump (400 W motor, model W2V10, WSA Co., Ltd., Gunpo, Korea). Before use, the filter cups were sterilized with 10% bleach followed by 70% ethanol and covered with a 1-mm nylon mesh to prevent the collection of macro-organisms. The two devices were positioned horizontally, approximately 0.5 m apart, on the balcony of the Biology Building. Air was sampled for 3 h per collection at a flow rate of 100 L/min, yielding a total air volume of 36 m^3^ (18 m^3^ per device). No microbial contamination was detected on blank filters. Aerosol samples were collected every 3–5 days throughout the study period, with occasional weather-related interruptions (e.g., during monsoon events), resulting in a total of 315 samples. The average annual temperature and precipitation during the sampling period were 15.6°C and 1,676 mm, respectively (KMA).

### DNA extraction

After sampling, the two filters were cut into small pieces using sterilized scissors and transferred into a 2 mL tube provided by the NucleoSpin Soil Kit (Macherey-Nagel, Düren, Germany). DNA extraction was performed according to the manufacturer’s instructions, with a minor modification. Specifically, prior to the lysis step, samples were sonicated for 30 min at 60°C (40 kHz) in a 10 L ultrasonic bath (Daihan Scientific Co., Ltd., Wonju, Korea). The extracted DNA was eluted in 50 µL of elution buffer and quantified using a NanoDrop 2000 spectrophotometer (Thermo Fisher Scientific Inc., Waltham, USA).

### Quantification of the 16S rRNA gene

Bacterial populations were quantified using qPCR targeting the 16S small subunit (SSU) rRNA gene, with the primer pair 340F (5′-TCCTACGGGAGGCAGCAG-3′) and 805R (5′-GACTACHVGGGTATCTAATCC-3′) (46, 47). Each qPCR (20 µL total volume) contained 2 μL of 10× PCR buffer (Cancerrop Inc., Seoul, Korea), 2 µL of 2 mM dNTPs, 0.4 µL of forward primer (10 µM), 0.8 µL of reverse primer (10 µM), 1 µL of 5× Invitrogen SYBR (Thermo Fisher Scientific), 2 units of Taq DNA polymerase (Cancerrop), and 1 µL of template DNA. PCR was initiated with a denaturation step at 95°C for 2 min, followed by 40 cycles of 95°C for 30 s, 50°C for 30 s, and 72°C for 30 s, with an additional 30-s reading step at 82°C. Reactions were run on an Applied Biosystems StepOnePlus real-time PCR system (Thermo Fisher Scientific). The quantification of qPCR results was expressed as SSU rRNA gene copy numbers per m^3^ of air.

### rRNA gene compositional analysis

Multiplex Illumina MiSeq sequencing was conducted using the 340F and 805R primer pair. The first round of PCR employed composite primers with overhang adapters (underlined): the forward primer 5′-TCGTCGGCAGCGTCAGATGTGTATAAGAGACAG-[340F]-3′ and the reverse primer 5′-GTCTCGTGGGCTCGGAGATGTGTATAAGAGACAG-[805R]-3′, with primer annealing at 50°C for 30 s, as described by Jeong, Choi (48). PCR products were purified using a FavorPrep PCR purification kit (Favorgen Corp., Pingtung, Taiwan), eluted in 30 µL of elution buffer, and quantified using a NanoDrop 2000 spectrophotometer (Thermo Fisher Scientific). The second round of PCR was conducted, as described by Jeong, Choi (48). The resulting PCR products were purified and quantified using the same methods, as described previously. The purified PCR products were pooled in equal amounts. MiSeq sequencing was performed by Macrogen Inc. (Seoul, Korea).

### Sequence processing and taxonomic assignment

Of the 315 aerosol samples collected, 293 yielded successful sequencing results. Data processing was performed using the QIIME2 pipeline (https://qiime2.org), which included quality filtering, chimera removal, and determination of amplicon sequence variants (ASVs). ASVs were taxonomically assigned using the SILVA SSU reference library (version 138). Non-bacterial sequences, including those assigned to Eukarya and Archaea, were excluded, resulting in an average final sequencing depth of 18,014 ± 11,922 reads per sample.

### Bacterial abundance

Bacterial populations were quantified by integrating absolute gene copy numbers from qPCR with relative abundances obtained from sequencing data. While sequencing provides the relative composition of 16S rRNA gene variants, qPCR quantifies their total abundance. Relative abundances of bacterial ASVs (excluding chloroplast and mitochondrial sequences) were scaled by multiplying them by the corresponding total 16S rRNA gene copy numbers from qPCR, producing an estimate of absolute bacterial abundance for each sample. The resulting data were log-transformed prior to statistical analyses. For 22 samples lacking sequencing data, bacterial populations were estimated by interpolation. Specifically, for 19 samples collected between 30 May and 11 August 2019, values were assigned based on the average from corresponding periods in subsequent years, reflecting consistent seasonal patterns (Fig. 1b). Linear interpolation was applied to estimate values for the remaining three samples.

### Meteorological and pollutant data

Meteorological variables (e.g., temperature, humidity) were obtained from the KMA, PM concentrations, which served as indicators of dust loading, were retrieved from the Korea Environment Corporation (https://www.airkorea.or.kr/web/). Metal concentrations within PM_10_ were acquired from the public database of Busan Metropolitan City (https://www.busan.go.kr/index). PM and metal concentration data from three monitoring stations near the sampling site were averaged to represent local conditions.

Dust levels in desert regions of China and Mongolia were assessed using air quality index (AQI) data collected from monitoring stations near Horqin Sandy Land (Chifeng, Siping, Chaoyang), Gobi Desert (Wuwei, Baotou, Jiayuguan, Zhangjiakou), and Taklimakan Desert (Aksu, Kumul), accessed via www.aqicn.org. AQI values were converted to PM_10_ mass concentrations using the calculation method described by Kumar and Goyal (49). Daily averages were computed for each desert region.

### Air mass transportation pathways

Origins of air masses were identified using back trajectory analysis with the NOAA Hybrid Single Particle Lagrangian Integrated Trajectory (HYSPLIT) model (https://www.ready.noaa.gov/HYSPLIT_traj.php) (50). For each sampling day, a 72-h backward trajectory was generated using the NCEP/NCAR Reanalysis data set, with an arrival height set at 10 m above ground level. The region surrounding the sampling site was divided into five sub-regions based on established pollutant source-tracking criteria (51) (Fig. S1). Air mass trajectories were visually inspected and assigned to one of these sub-regions.

### Data analysis

All statistical analyses were performed using R software (version 4.3.0; R Core Team). To reduce temporal noise, data were initially aggregated into weekly time series. Multicollinearity among environmental variables was evaluated using correlation analysis (*psych* package), and variables with correlation coefficients greater than 0.8 were excluded from further analyses. For example, temperature was highly correlated with absolute humidity, dew point, vapor pressure, surface temperature, and minimum grass temperature; relative humidity with absolute humidity, dew point, and vapor pressure; and solar radiation with large and small evaporation.

A correlation-based network analysis was employed to identify environmental factors associated with bacterial abundance. Monthly-aggregated data were used to calculate Spearman’s correlation coefficients among a range of variables (*psych* package), including bacterial abundance, desert PM_10_, local PM_10_, meteorological factors (temperature, atmospheric pressure, precipitation, wind velocity, relative humidity, solar radiation, ultraviolet, cloud, sunshine, and diffusion), pollutants (metals: Al, As, Ca, Cd, Cr, Cu, Fe, Mg, Mn, Ni, Pb; gases: CO, SO_2_, NO_2_, O_3_), traffic volume, and air mass pathways (A–E). Statistically significant correlations (*P* < 0.05) were visualized using Cytoscape (version 3.8.2). To emphasize key relationships involving bacterial abundance, desert PM_10_, and local PM_10_, intercorrelations among other environmental variables were omitted from the final network.

The impacts of Asian dust events on bacterial abundance were assessed by comparing bacterial levels before and during official Asian dust events and PM_10_ spikes. According to the KMA, official Asian dust events are defined as periods when PM_10_ concentrations exceed 150 µg·m^−3^ for more than two consecutive hours, associated with air masses originating from the Gobi and Taklimakan deserts. In this study, PM_10_ spikes were defined as instances when concentrations exceeded 45.0 µg·m^−3^ (approximately 150% of the study’s average), originating from the same desert regions.

Annual cycles of bacterial abundance, desert PM_10_, and local PM_10_ were analyzed using parametric modeling. Missing weekly data points were imputed using a weighted moving average: *x*_t_ = 1/6 × *x*_*t* − 3_ + 1/3 × *x*_*t* − 2_ + 1/2 × *x*_*t* − 1_, to preserve temporal continuity. The same method was applied to outlier correction. Each time series was modeled using a sine/cosine function (*Y* = *a‧sin*(2‧π‧*t*) + *b‧cos*(2‧π‧*t*)), capturing predominant seasonal patterns. Spectral analysis and time-series decomposition (*stats* package) were also conducted to identify periodicities underlying the time series and support their interpretation.

SEM was performed to quantitatively examine the combined effects of LA generation and RD on airborne bacterial dynamics. The analysis was performed (*lavaan* package) with the original data used following the removal of outliers. The SEM framework included three latent variables: LA, LG, and RD. LA was represented by bacterial abundance and local PM_10_; LG by humidity, atmospheric pressure, and cloud cover; and RD by dust immigration from the three desert regions. Dust immigration was estimated by multiplying desert PM_10_ concentrations by the frequency of air mass pathway A (based on HYSPLIT outputs at 2-h intervals), and the resulting values were smoothed using a 4-day moving average. Model fit was evaluated using standard indices: TLI, RMSEA, and SRMR.

## Data Availability

All sequence data have been deposited in the DNA Data Bank of Japan Sequence Read Archive (https://www.ddbj.nig.ac.jp/dra) under accession number DRA016519. The data underlying this article will be shared on request to the corresponding author.

## SUPPLEMENTAL MATERIAL

Supplemental material aem.01345-25-s0001.docx Table S1; Fig. S1 to S6.

ASM does not own the copyrights to Supplemental Material that may be linked to, or accessed through, an article. The authors have granted ASM a non-exclusive, world-wide license to publish the Supplemental Material files. Please contact the corresponding author directly for reuse.
