# Supplementary material for: Annual patterns of airborne bacteria governed by local generation and regional dispersal
Source: Appl Environ Microbiol. 2026 Jan 28;92(2):e01345-25. doi: 10.1128/aem.01345-25 (PMC12915337; doi:10.1128/aem.01345-25)
Supplement: Supplemental material — Table S1; Fig. S1 to S6. [file aem.01345-25-s0001.docx]

Supplementary results for

Annual patterns of airborne bacteria governed by local generation and regional dispersal

So-Yeon Jeong, Chi Won Lee and Tae Gwan Kim*

Department of Microbiology, Pusan National University, Pusan 46241, Korea

*Corresponding Author: Tae Gwan Kim

Tel: +82 51 510 2268; Fax: +82 51 514 1778

E-mail address: [tkim@pusan.ac.kr](mailto:tkim@pusan.ac.kr)

Table S1. Most abundant bacterial taxa at the family and genus levels detected during Asian dust events and PM_10_ spikes. The top 16 families and 30 genera (by relative abundance) were selected to enable comparison with previously reported source‑region microbiota (Fig. S2). Taxa are listed alphabetically within each rank.

| No. | Taxonomy | No. | Taxonomy |
| --- | --- | --- | --- |
|  | Family |  | Genus |
| 1 | Acetobacteraceae | 1 | *Acinetobacter* |
| 2 | Bacillaceae | 2 | *Arthrobacter* |
| 3 | Beijerinckiaceae | 3 | *Bacillus* |
| 4 | Burkholderiaceae | 4 | *Blastococcus* |
| 5 | Chitinophagaceae | 5 | *Cellulomonas* |
| 6 | Chroococcidiopsaceae | 6 | *Chroococcidiopsis* |
| 7 | Geodermatophilaceae | 7 | *Clostridium* |
| 8 | Hymenobacteraceae | 8 | *Craurococcus* |
| 9 | Micrococcaceae | 9 | *Friedmanniella* |
| 10 | Moraxellaceae | 10 | *Gemmatirosa* |
| 11 | Nocardiaceae | 11 | *Geodermatophilus* |
| 12 | Nocardioidaceae | 12 | *Hymenobacter* |
| 13 | Planococcaceae | 13 | *Kocuria* |
| 14 | Rhizobiaceae | 14 | *Massilia* |
| 15 | Rhodobacteraceae | 15 | *Methylobacterium* |
| 16 | Sphingomonadaceae | 16 | *Microvirga* |
|  |  | 17 | *Modestobacter* |
|  |  | 18 | *Nocardioides* |
|  |  | 19 | *Noviherbaspirillum* |
|  |  | 20 | *Ochrobactrum* |
|  |  | 21 | *Paracoccus* |
|  |  | 22 | *Pseudarthrobacter* |
|  |  | 23 | *Rhodococcus* |
|  |  | 24 | *Romboutsia* |
|  |  | 25 | *Rubellimicrobium* |
|  |  | 26 | *Segetibacter* |
|  |  | 27 | *Skermanella* |
|  |  | 28 | *Sphingomonas* |
|  |  | 29 | *Staphylococcus* |
|  |  | 30 | *Streptomyces* |


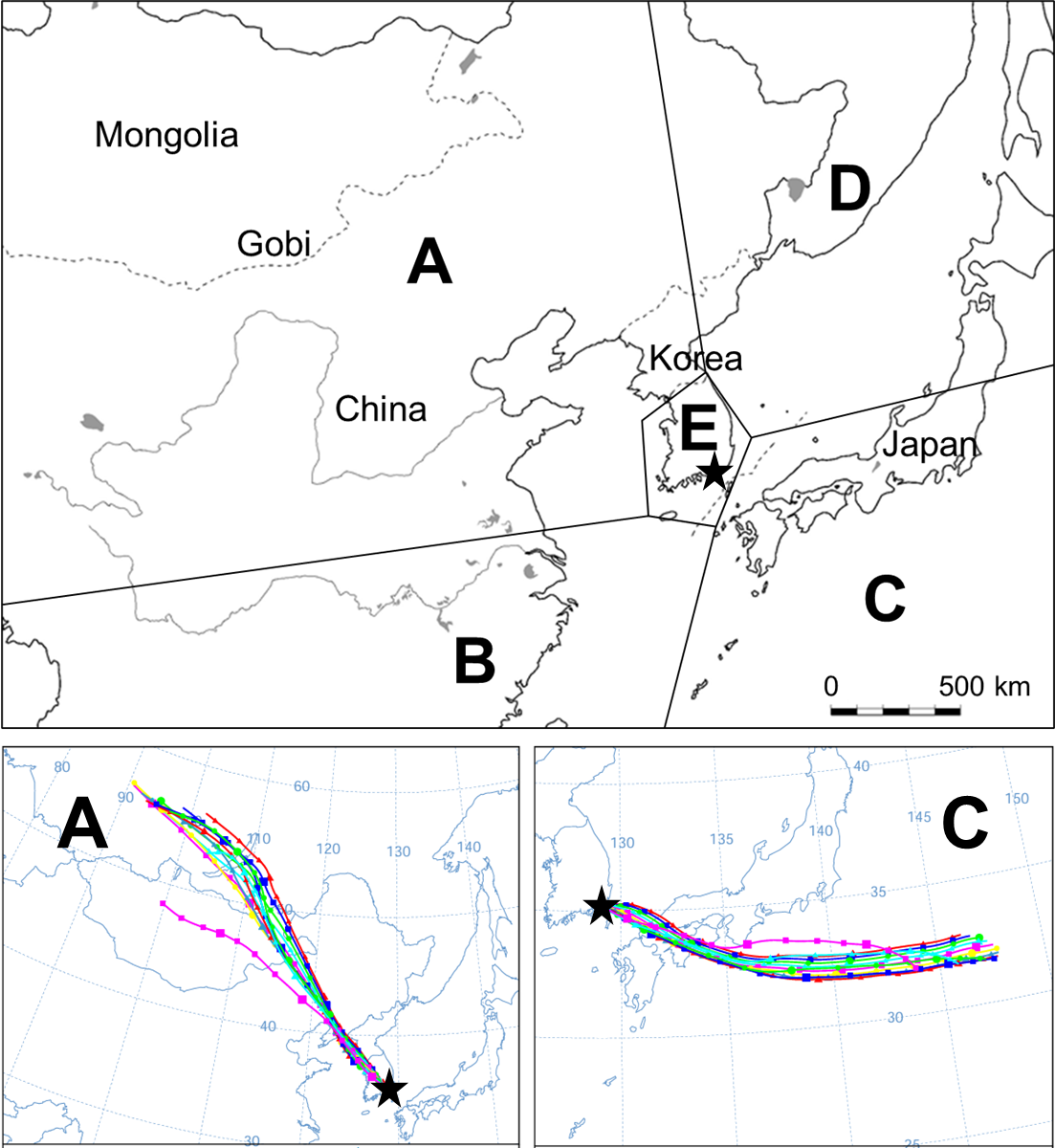


Fig. S1. The five classified pathways of air mass transport in this study. Pathway A: air masses passing from northern and mid-western parts of China; B: passing from southern parts of China; C: passing from North Pacific Ocean and southern parts of Japan; D: passing from the East Sea and northern parts of Japan; and E: local retained on South Korea. A representative map illustrates example trajectories for pathways A (March 6, 2022) and C (July 18, 2021), generated using the NOAA HYSPLIT model.


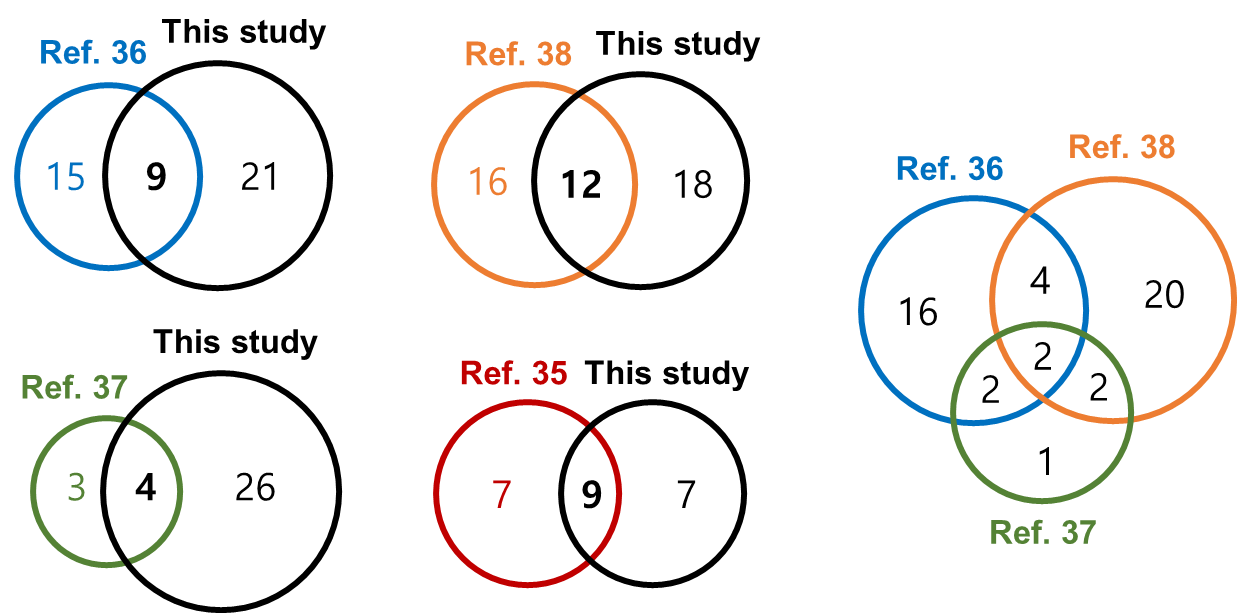


Fig. S2. Venn diagrams showing the overlap of dominant bacterial taxa between source‑region bacterial communities reported in previous studies and airborne communities detected in Busan during Asian dust events and PM_10_ spikes (see Table S1). References 36–38 present genus‑level comparisons; reference 35 presents a family‑level comparison. Numbers denote the counts of shared and unique dominant taxa.

Fig. S3. Spectral analysis plot. Spectral density was plotted against the time frequency (year).

Fig. S4. Time-series decomposition of airborne bacterial population. (a), observed data and trend component; (b), seasonal component; and (c), random component.

Fig. S5. Time-series decomposition of local PM_10_. (a), observed data and trend component; (b), seasonal component; and (c), random component.

Fig. S6. Time-series decomposition of desert dust PM_10_. (a), observed data and trend component; (b), seasonal component; and (c), random component.
